# Role of the Pangolin in Origin of SARS-CoV-2: An Evolutionary Perspective

**DOI:** 10.3390/ijms23169115

**Published:** 2022-08-14

**Authors:** Shishir K. Gupta, Rashmi Minocha, Prithivi Jung Thapa, Mugdha Srivastava, Thomas Dandekar

**Affiliations:** 1 Evolutionary Genomics Group, Center for Computational and Theoretical Biology, University of Würzburg, 97074 Würzburg, Germany; 2 Functional Genomics & Systems Biology Group, Department of Bioinformatics, Biocenter, Am Hubland, University of Würzburg, 97074 Würzburg, Germany; 3 Department of Biochemistry, All India Institute of Medical Sciences, New Delhi 110029, India; 4 Core Unit Systems Medicine, University of Würzburg, 97080 Würzburg, Germany; 5 EMBL Heidelberg, BioComputing Unit, 69117 Heidelberg, Germany

**Keywords:** COVID-19, SARS-CoV-2, origin, evolution, intermediate host, pangolin, mutation, recombination, adaptation, transmission, comparative sequence analysis

## Abstract

After the recent emergence of SARS-CoV-2 infection, unanswered questions remain related to its evolutionary history, path of transmission or divergence and role of recombination. There is emerging evidence on amino acid substitutions occurring in key residues of the receptor-binding domain of the spike glycoprotein in coronavirus isolates from bat and pangolins. In this article, we summarize our current knowledge on the origin of SARS-CoV-2. We also analyze the host ACE2-interacting residues of the receptor-binding domain of spike glycoprotein in SARS-CoV-2 isolates from bats, and compare it to pangolin SARS-CoV-2 isolates collected from Guangdong province (GD Pangolin-CoV) and Guangxi autonomous regions (GX Pangolin-CoV) of South China. Based on our comparative analysis, we support the view that the Guangdong Pangolins are the intermediate hosts that adapted the SARS-CoV-2 and represented a significant evolutionary link in the path of transmission of SARS-CoV-2 virus. We also discuss the role of intermediate hosts in the origin of Omicron.

## 1. Introduction

Since the beginning of the coronavirus disease 2019 (COVID-19) pandemic in 2019, as of 26 June 2022, there have been approximately 574 million confirmed cases of COVID-19 worldwide that have claimed the lives of nearly 6.5 million individuals (weekly epidemiological update on COVID-19, see link: who.int (accessed on 3 August 2022)). The causative agent of COVID-19 has been identified as a novel coronavirus named severe acute respiratory syndrome coronavirus-2 (SARS-CoV-2). The outbreak of this disease has caused a massive disruption in the global economy, leaving many developing as well as developed nations in financial turmoil. The rollout of different vaccines has eased the pressure of increasing infection rates. However, the emergence of new variants of SARS-CoV-2 is posing more uncertainty on the level of devastation that may occur in the future. Hence, there is a clear need for in-depth understanding of SARS-CoV-2’s evolutionary mechanisms in order to prepare better for zoonotic transmissions and potential pandemics of the future. There is an overwhelming consensus regarding the origin of SARS-CoV-2 from bats. However, its evolutionary history and path of transmission still remains elusive and thus, a better understanding of the cross-species transmission and evolutionary relationship between SARS-CoV-2 and other highly related coronaviruses (CoVs) is required.

The SARS-CoV-2 virus enters into the host cell through a two-step mechanism: (i) host-cell receptor recognition and (ii) host-virus cell membrane fusion. First, the receptor-binding domain (RBD) of the spike (S) glycoprotein in coronavirus SARS-CoV-2 is employed to interact with the functional angiotensin converting enzyme 2 (ACE2) receptors that are known to be expressed in many human organs [1]. ACE2 has also been recognized as a receptor for human coronavirus HCoV-NL63 and SARS-CoV [2]. Second, this ACE2 receptor recognition by the S-glycoprotein of SARS-CoV-2 leads to significant changes in the conformation of S-glycoprotein, consequently causing a fusion of host–virus cell membrane. In order to fight against the SARS-CoV-2 infection, the host cell generates neutralizing antibodies against the S-glycoprotein. Several research labs are attempting to understand how the virus escapes from neutralization. Thus, a systemic understanding of the RBD-ACE2 interface may provide important insights for such a study. This will also help us to determine active regions in the S-glycoprotein-ACE2 interface that can act as potential targets for designing SARS-CoV-2 drugs based on blocking of the S-glycoprotein-ACE2 interaction. Notably, identification of critical amino acid positions involved in this interaction will further provide us significant information on the evolutionary emergence of SARS-CoV-2 and may answer several outstanding questions related to the origin and path of transmission of SARS-CoV-2.

## 2. Current Understanding on the Intermediate Host of SARS-CoV-2 and Our Perspective

Since the outbreak of SARS-CoV-2, several independent groups have attempted to shed light on its zoonotic origin and emergence. For instance, bat coronavirus (Bat-CoV) RaTG13, a coronavirus isolated from the bat species *Rhinolophus affinis* obtained from Yunnan Province, has been found to share the closest whole-genome identity of 96% with SARS-CoV-2 [3]. In another study, cryo-EM structures of spikes of CoVs RaTG13 and GX Pangolin-CoV isolated from bat and pangolin species, respectively, were found to be closely related to the SARS-CoV-2 spike structure [4]. Furthermore, sequence differences of the SARS-CoV-2 spike proteins, its ACE2 interacting efficiencies and pathophysiological responses in humans and bats indicate the circulation and evolution of progenitor virus in single or multiple intermediate hosts, as in the previously existing coronaviruses SARS-CoV and MERS-CoV, where the progenitor virus passed through hosts such as minks, camels, etc., before jumping to humans [5,6,7]. The natural and intermediate hosts co-evolve with the virus and act as reservoirs for the virus replication [8]. Recombination and/or horizontal gene transfer with other closely related viruses inside the intermediate hosts can lead to the virus gaining additional survival and pathogenic features [5].

An intermediate host provides a selective environment that allows a zoonotic virus, not only to multiply massively in a second host but also to acquire mutations and evolve so as to become compatible with new hosts. Thus, it is critical to recognize the intermediate host as a dangerous virus reservoir for a new viral outbreak, and by interfering with viral replication in the intermediate host the viral transmission chain can be disrupted, and further spread can be controlled. Identifying intermediate host species not only permits the introduction of risk mitigation public health strategies, but also offers a deeper understanding of the evolution and pathogenesis of zoonotic diseases. According to our current understanding on the transmission chain of SARS-CoV2, bat is its original host while pangolin is likely its intermediate host, although details of the complete transmission process are still unaddressed [9]. Considering the importance of the intermediate host, two independently published *Nature* articles, first by Lam et al. (2020) [10] and then the work of Xiao et al. (2020) [11], provided a strong statement that Malayan pangolins are the potential intermediate hosts in the emergence of the novel coronavirus: Xiao et al. (2020) [11] reported the presence of a SARS-CoV-2-like virus (named “Pangolin-CoV”) in 17 out of 25 Malayan pangolins collected from Guangdong province in South China. CoV-infected pangolins showed clinical symptoms of the disease, and 14 infected animals died within a span of 1.5 months [11]. By analyzing the RNAseq data of lung samples from pangolins followed by sequence comparisons, the authors demonstrated high sequence identity between Pangolin-CoV and SARS-CoV-2 genes.

Regarding the paper by Xiao et al. (2020) [11], we argue that the article did not focus well to present critical residues in their CoVs sequence comparison (provided in their Figure 7b), and in consequence, we hereby now show new in our alignment how and where amino acid substitutions occur in the host ACE2 receptor. These ACE2 residues are interacting with important residues of the receptor-binding domain (RBD) of spike glycoprotein (S) in the coronavirus. We show and compare for these isolates from bats and virus isolates from pangolins collected from Guangdong province (GD Pangolin-CoV) and the Guangxi autonomous region (GX Pangolin-CoV) in South China.

## 3. Sequence Comparative Analysis of Coronavirus Isolates from Bat and Pangolins

To validate the CoVs multiple sequence alignment (MSA), we retrieved the exact sequences as used in the analysis of Xiao et al. (2020) [11] and performed genome annotation of GD Pangolin-CoV (GISAID accession ID: EPI_ISL_410721) with VIGOR [12] to extract S-protein sequences.

**Figure 1:**
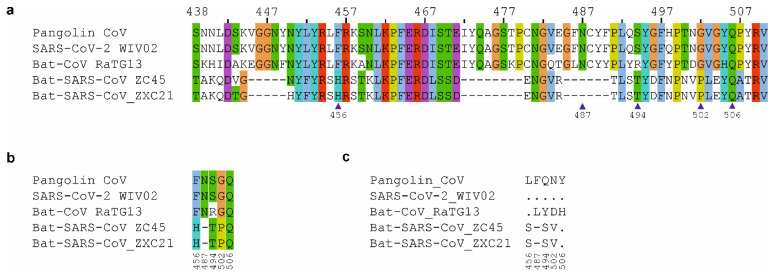
**Analysis of the RBD sequence**. (**a**) Multiple sequence alignment showing RBD of S protein from Pangolin CoV (GISAID accession ID: EPI_ISL_410721), SARS-CoV-2_WIV02 (EPI_ISL_402127), and Bat-CoVs (Bat-CoV_RaTG13, Bat-SARS-CoV_ZC45 and Bat-SARS-CoV_ZXC21). Conserved similar amino acids are colored in the alignment. The five critical residues for human ACE2 binding (as mentioned in [11] with respect to SARS-CoV-2_WIV02 coordinates) are indicated by arrowheads below the alignment. Sequence gaps are indicated with dashes. (**b**) Central part of the alignment from (**a**) showing the critical residues. (**c**) Part of alignment from Xiao et al. [11] Figure 7b. This shows only the mentioned critical residues. Identical residues are indicated with a dot.

Other S-protein sequences from human isolate SARS-CoV-2_WIV02 and bat isolates (Bat-CoV_RaTG13, Bat-SARS-CoV_ZC45 and Bat-SARS-CoV_ZXC21) were retrieved from GenBank. We used L-INS-I, the most accurate algorithm of their software suite “MAFFT” for MSA [13]. Out of the five suggested positions (456, 487, 494, 502 and 506), we found that both in SARS-CoV-2_WIV02 and GD Pangolin-CoV, these positions correspond to Phe, Asn, Ser, Gly and Gln, respectively (Figure 1a,b). The differences found in critical positions from Xiao et al. (2020) [11] are depicted in Figure 1b,c. Moreover, the residues we found at these critical positions are in direct agreement with other recent studies [10,14,15].

**Figure 2:**
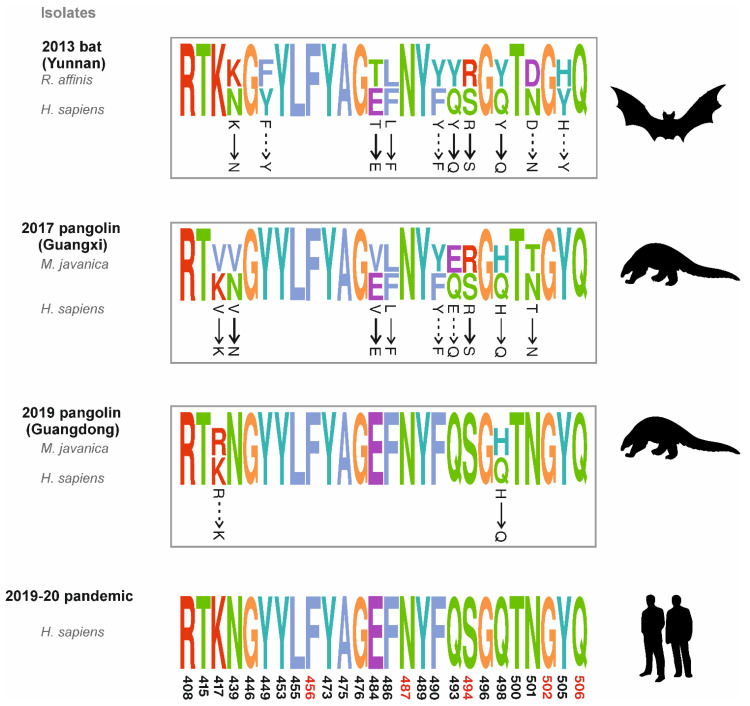
**Detailed comparison of ACE2 binding RBD residues of Bat-CoV_RaTG13, Pangolin CoVs and SARS-CoV-2.** The level of conservation is visualized using seqLogo, the letter height is proportional to the level of conservation of each residue in the sequence and the color code indicates similar physicochemical attributes. Residues important for ACE2 binding were obtained from [14,15,18] and mapped to the Wuhan-Hu-1 referenced SARS-CoV-2 S protein sequence (GISAID accession ID: EPI_ISL_402125). To avoid visual misinterpretation, in each block equal number of sequences from the species in comparison were taken. In the seqLogo graphic wherever two amino acids are aligned, SARS-CoV-2 sequence conservation is shown at lower position. Required mutations in bat and pangolin RBD for human adaptation are shown with arrows (Normal arrow: semi-conservative substitution; Dashed arrow: non-conservative substitution and Bold arrow: conservative substitution). Critical residue described in Xiao et al. [11] is marked in red color at the bottom of the figure.

The receptor binding domain (RBD) is an important and highly relevant target for identification of neutralizing antibodies and vaccine development [16]. There is a wide mutational space in RBD that can maintain proper folding, expression and generation of consistent phenotypes in SARS-CoV-2 [17]. Recent studies have identified 26 important RBD residues [14,15,18] that are involved in the interaction with the ACE2 receptor and collected more samples [10,19] of CoV-like viruses being isolated from pangolin species. Using information from these analyses, we offer an extension to the study by Xiao et al. (2020) [11] to illuminate the role of pangolins in the origin of SARS-CoV-2. To this end, we mapped all the residue positions according to the Wuhan-Hu-1 reference sequence (GISAID accession ID: EPI_ISL_402125). We observed an interesting trend of mutations here (Figure 2): *Rhinolophus affinis* isolated Bat-CoV_RaTG13 in 2013 needs at least four non-conservative and two semi-conservative mutations in RBD for human adaptation. Pangolin-CoV isolated from Guangxi [10] in 2017 needs three non-conservative and three semi-conservative mutations for human adaptation. Notably, Pangolin-CoV isolated from Guangdong [11,19] in 2019 requires only one semi-conservative mutation for human adaptation. The calculation of binding energy between soluble human ACE2 and RBD both in the SARS-CoV-2 and pangolin isolated CoVs confirmed that GD Pangolin-CoV binds comparatively more strongly to the ACE2 receptor than SARS-CoV-2 [11,17]. This provides first clear evidence that as compared to the GX Pangolin-CoV, the GD Pangolin-CoV is more likely to be involved in the origin of SARS-CoV-2.

Furthermore, in the sequence of Bat-CoV_RaTG13, we observed Arg at position 494 while the other four critical residues highlighted in [11] were homologous to that of SARS-CoV-2_WIV02 and GD Pangolin-CoV (Figure 1b). GX Pangolin-CoV also had Arg at position 494, which was found to be mutated into Ser in Guangdong pangolins (Figure 2). It is noteworthy that Ser494 strengthens the structural stability of the virus-binding hotspot Lys353 at ACE2 surface [18,20]. On the contrary, [11] reported Gln at position 494 in GD Pangolin-CoV (Figure 1c).

In the sequence of GD Pangolin-CoV, at position 498, Glu was observed while in SARS-CoV-2, His was identified at the same position. Reverting this mutation (His to Glu) at position 498 in SARS-CoV-2 causes a 5-fold increase in its human ACE2 binding affinity as compared to the wild type [21]. Although SARS-CoV-2 is not known to infect porcine cells, a 12-fold increase in infection efficiency in porcine cell lines was observed with Q498H mutation in SARS-CoV-2. Another study showed that when the Q498H mutation was introduced to SARS-CoV-2 RBD, an enhancement of its binding capacity to ACE2 homologs in mouse, rat and European hedgehog was observed, suggesting that pangolin CoVs are also capable of infecting humans [21]. In the sequence of GD Pangolin-CoV, at position 498, Glu was observed while in SARS-CoV-2, His was identified at the same position. Reverting this mutation (His to Glu) at position 498 in SARS-CoV-2 causes a 5-fold increase in its human ACE2 binding affinity as compared to the wild type [21]. Although SARS-CoV-2 is not known to infect porcine cells, a 12-fold increase in infection efficiency in porcine cell lines was observed with Q498H mutation in SARS-CoV-2. Another study showed that when the Q498H mutation was introduced to SARS-CoV-2 RBD, an enhancement of its binding capacity to ACE2 homologs in mouse, rat and European hedgehog was observed, suggesting that pangolin CoVs are also capable of infecting humans [21].

In porcine cell lines, mutation in the amino acid position 498 from Glu to His in GD Pangolin-CoV and GX Pangolin-CoV displayed significant reduction in infectivity with human ACE2 by more than 200-fold as well as 160-fold, respectively [21,22]. In addition, mutation at the same residue 498 from Glu to Tyr in Bat-CoV has been shown to enhance the binding affinity of RBD to ACE2 [17]. These observations confirm that the Q498H mutation in SARS-CoV-2 spike could be a potential threat to humans. Both GD Pangolin-CoV and SARS-CoV-2 with the Q498H spike mutation may infect farm pigs. Hence, this high potential of an outbreak in farm pigs and meat industry indicates vice versa also a clear hazard of reverse anthropo-zoonotic infections. Luckily, the Q498H mutation in SARS-CoV-2 strains is not widespread yet and at the time of writing this article, only 78 strains with spike Q498H mutations have been submitted to the GISAID database [23] as of 7 August 2022. These strains were identified from Europe (36), North America (19), Asia (14), Africa (4), Oceania (4), South America (1). Thus, surveillance of such a host range expansion associated mutation is extremely critical as it may lead to a zoonotic transmission between pangolin–human–pig, which may lead to a new type of zoonotic epidemic [21]. Notably, Q498H mutation is also found in coronavirus isolated from Laos horseshoe bats (*R. malayanus* (virus BANAL-52), *R. marshalli* (BANAL-236), *R. pusillus* (BANAL-103)) that has recently been identified as the closest relative of SARS-CoV-2 [24]. These viruses have >95% identity at sequence level to SARS-CoV-2 [25]. Moreover, out of more than 40 mutations in spike proteins, the current SARS-CoV-2 strain of concern Omicron contains Q498R and K417N. Interestingly, Bat-CoV_RaTG13, Beta (B.1.351 lineage) and Delta variant of SARS-CoV-2 consists of K417N. K417N mutation is known to increase the ACE2 binding and escape binding by SARS-CoV-2 neutralizing monoclonal antibodies (mAbs) such as LY-CoV016 (etesevimab) and C105 [26,27].

## 4. Role of Genomic Recombination between Bat and Pangolin Coronaviruses

It was identified by Li et al. (2020) that the GD Pangolin-CoV clade was more similar (91.2%) to SARS-CoV-2 than the GX Pangolin-CoV clade (85.4%) [28]. As compared to Bat-CoV, despite its high identity region in the RBD region, GD Pangolin-CoV showed reduced overall sequence similarity compared to SARS-CoV-2. This suggests that the SARS-CoV-2 may be originated by possible recombination events primarily between GD Pangolin-CoV and Bat-CoV RaTG13 or other related CoV’s. Recombination is one of the most widespread mechanisms by which virus genomes are known to evolve and adapt themselves to new hosts and new environments. Recombination in viruses occurs when two or more viruses co-infect the same host organism and exchange part of their genetic segments by “crossover” of their nucleic acid strands. In fact, there is growing evidence for recombination events in viruses to be associated with genetic variability, virus adaptation, new virus emergence, increased virulence, alteration of transmission mechanisms and rates, escape from host immune response and increased resistance to anti-viral drugs [29,30]. Thus, understanding patterns and mechanisms of recombination in viruses will help us to identify potential risks associated with virus adaptation. Recombination events between CoVs from different hosts are being studied in order to explore their role in the origin of SARS-CoV-2 [31]. It was found that recombination is one of the crucial factors contributing to the diversity of beta coronaviruses, and these viruses tend to have recombination in high frequencies [32]. There is strong evidence of purifying selection and recombination of bat-CoV with different CoVs along multiple hosts that led to the evolution on present SARS-CoV-2 [28,33]. In one study, three independent recombination events were identified from SARS-CoV-2 strains and proximal outgroups using the RDP4 algorithm. It was hypothesized that two of these recombination events could have altered the two Pangolin-CoVs. We see routes to two isolates, one that evolved from GX Pangolin-CoV and another from GD Pangolin-CoV. These events also indicate that some fragments in the SARS-CoV-2, Bat-CoV-RaTG13 and Pangolin-CoVs were possibly integrated from bat-CoV ZC45-ZXC21 through recombination [26]. Another study using horizontal gene transfer and recombination analysis revealed statistically significant recombination events between RaTG13 and GD Pangolin-CoV in S and N genes [33]. Additionally, more recombination events have been observed in ORF1ab, S, ORF3a, ORF7a, ORF8 and N genes between ancestors of SARS-CoV-2, RaTG13, GD Pangolin CoV and bat-CoV ZC45-ZXC21 [33]. As bat-CoV is able to infect pangolins, pangolins could be a source and reservoir for gene transfer and recombination between RaTG13 and GD Pangolin-CoV as well as other related CoVs [33]. SARS-CoV-2 and RaTG13 share the highest overall sequence similarity in the S gene. But when focusing specifically on the variable loop of the S-gene, a stronger extent of sequence similarity is observed between SARS-CoV-2 and GD Pangolin-CoV compared to SARS-CoV-2 and RaTG13. This might suggest acquisition of a new RBD sequence from the recombination event between the common ancestral lineage of SARS-CoV-2 and RaTG13. Alternatively, this happened after the lineages leading to the SARS-CoV-2 and RaTG13 split with GD Pangolin-CoV, including further subsequent mutations leading to variations in the RBD [34]. It was also shown that the RaTG13 pseudo viruses are unable to properly bind to ACE2 expressing cells, which means that the recombination event in the S region could have led to the SARS-CoV-2 last ancestral lineage to jump to humans as there is a difference of only one non-conservative mutation between RBD of SARS-CoV-2 and GD Pangolin-CoV [15]. Additionally, the antigenicity of GD Pangolin-CoV is very close to SARS-CoV-2 as compared to that of RATG13 and GX Pangolin-CoV. The GD Pangolin-CoV pseudo-typed viruses are neutralized by the neutralizing antibodies produced against SARS-CoV-2, the vaccine-induced serum and convalescent sera from COVID-19 patients with higher activity than the normal SARS-CoV-2 viruses themselves, whereas in the cases of RATG13 and GX Pangolin-CoV, poor cross-neutralization has been observed. We speculate that the RBD of SARS-CoV-2 has emerged by a combination of incremental adaptive mutations. Our observations strongly support the high identity of RBD of SARS-CoV-2 with Guangdong Pangolin-CoV but not with Guangxi pangolins. This further indicates that from Guangdong Pangolin-CoV to SARS-CoV-2 evolution, only few adaptations are required for ACE2 binding. Of note, geographically in comparison to Guangxi, Guangdong is closer to Wuhan.

## 5. Discussion

Amidst the mad rush of publications addressing the key question of the transmission of SARS-CoV-2, finding the source of intermediate hosts and involvement of several new hosts will be an essential contribution to study and understand the zoonotic COVID-19 outbreak. Moreover, the importance to consider all involved intermediate hosts applies to zoonotic diseases in general, particularly flu virus, coronavirus and Flaviviridae as well as the notorious vector-borne tropical diseases. Herein, we report the transmission process of SARS-CoV-2 from bats to humans through the pangolins, which can be regarded as an intermediate host for the novel coronavirus. In particular, as established by functional data for bats and pangolins, we can refute earlier publications questioning this and putting the whole zoonotic transition process just on bats as the sole culprit for transmission.

The evolutionary goal of a virus is to spread rather than to kill. To increase its transmission, the virus must reduce its infectivity or severity. If a virus is lethal, it will kill its host quickly, which will limit its ability to multiply and infect, which will ultimately [35] reduce its transmission. We speculate that the His to Glu mutation at the 498 position in SARS-CoV-2 is favorable for the virus to better reach this evolutionary optimum. In SARS-CoV-2 infected patients, the highest percentage of virus shedding, and thus transmissibility, occurs 1 to 2 days before the infected person begins to display symptoms [36]. It has been reported that 40–45% of SARS-CoV-2 infected people remain asymptomatic [37] and such carriers may be able to transmit the virus for a longer period. Altogether, these findings might explain the different SARS-CoV-2 evolutionary trade-offs. Furthermore, we illuminate the scenario that current SARS-CoV-2 originated by the recombination between Guangdong Pangolin-CoV and other Bat-CoV such as *R. malayanus* [38] or some yet to be identified Bat-CoVs that resulted in the addition of cleavage sites for human furin proteases. This plausible phylogeny emphasizes the importance of genomic recombination events in the evolution of these viruses and seconds previous studies that exclusively rely on and claim the highly recombinogenic nature of coronaviruses [39].

Of note, a host’s adaptive immune system also plays a contributing role in the evolution of a virus. Many recent studies have focused on understanding the involvement of intra-host infection dynamics in the emergence of new variants of SARS-CoV-2, especially in case of a chronic infection. For instance, a recent study identified several mutations in the SARS-CoV-2 genomic sequence that accumulated during persistent COVID-19 infection in an immunosuppressed patient [40]. Interestingly, the viral genotype was relatively stable during the first few weeks of infection, while most of the synonymous and non-synonymous mutations started accumulating following the 42nd day onward of persistent infection. In similar other case reports, researchers have demonstrated the emergence of novel multi-mutational viral variants in immunosuppressed patients with chronic viral infection [41,42,43]. It is noteworthy that the newly detected SARS-CoV-2 variants in the immunosuppressed patients showed a partial escape from vaccine-induced humoral immune response and thus could serve as the seeds for a new epidemic. It is also conceivable that the rate at which virus evolves in immunosuppressed COVID-19 patients is much higher than the community-driven rate of vial evolution [44]. Thus, immunocompromised individuals may serve as the secondary intermediate hosts responsible for an accelerated evolutionary jump, thereby posing an obstacle in the eradication of the COVID-19 pandemic.

The high number of spike mutations observed in the Omicron strain [45] again highlights the role of the intermediate host. Omicron’s origin from other human SARS-CoV-2 variants is questionable, as in a short time scale such a heavy number of mutations cannot be fixed by selection pressure. Regarding the origin of Omicron in humans, we consider as a most likely scenario that some chronically infected COVID-19 patient survived for a longer time period with the virus and provided thus a suitable host environment for the virus to mutate and increase adaptation. Phylogenetic analysis has suggested Omicron has evolved in parallel with other variants and diverged from other strains at least before mid-2020 [46,47]. This again indicates there should be a missing intermediate non-human animal host where the virus has accumulated mutations. A recent preprint suggests a possible mouse origin of Omicron [48].

By considering the current scenario of pandemic and transmission-mortality trade-off theory [49], we argue that evolutionary pressures do not make the virus more deadly, so we can expect the future variants of SARS-CoV-2 should be less deadly. Since the evolutionary pressure will favor over time more transmissible but less virulent strains, in the future, with fully vaccinated people, the pandemic will turn into a manageable infection such as common cold or flu. As there will always be the risk of a new virus causing a pandemic, better wildlife protection and restricted zoonotic disease exposure including pangolins will be critical in minimizing the risk of future zoonotic infection transmission.

The emergence of various variants of SARS-CoV-2 in recent times suggests that the virus population has a strong potential to evolve further in future. There is a possibility that genetic material of SARS-CoV-2 can recombine with genetic material of other coronavirus in animal hosts. Individuals with SARS-CoV-2 infections who come in close contact with coronavirus-infected animals can act as intermediate hosts for the development of recombinant viruses. Similarly, animals with coronavirus infections on meeting with SARS-CoV-2 infected individuals can act as intermediate hosts where recombinant strains can evolve for further species jump. If the recombinants are lethal, these can give rise to a new epidemic or pandemic. There is evidence that SARS-CoV-2 strains are already recombining with other SARS-CoV-2 strains [50,51]. With diversification of virulence evolution, the risk of multiple infections may also increase. In addition, the question is whether the first-generation vaccines for COVID-19 would be effective for the upcoming variants. There are many theories in this regard, and studies are still underway before anything can be said with confidence. Thus, it is very important to monitor closely the evolutionary trajectory or the evolutionary dynamics of SARS-CoV-2. Additionally, we have sequence datasets now available based on SARS-CoV-2 genomes [23] that can be used to determine if any specific sites in the viral genomic sequence are more prone to adaptive evolution. All these studies would help us to track the spread of the virus and thus efficiently manage public health strategies and therapeutic interventions for its control.

## 6. Conclusions

Since the first reports of SARS-CoV-2 virus infection came from Wuhan, there has been a considerable debate on its zoonotic origin and path of transmission among the human population. Through this article, we offer our perspective and an extension to the current understanding on the intermediate host for SARS-CoV-2. To this end, we performed sequence comparative analysis of the host ACE2-interacting residues of the RBD of spike glycoprotein in SARS-CoV-2 isolates from bats compared to the respective residues from pangolin isolated CoVs collected from Guangdong province and Guangxi autonomous regions of South China. Our analysis supports that Guangdong Pangolins are the intermediate hosts, which also provides the evidence that RBD of GD Pangolin-CoV has a stronger affinity for the ACE2 receptor as compared to the SARS-CoV-2. Further surveillance of GD Pangolin-CoV at a larger scale could help us to better understand their path of transmission in more detail. Additionally, it is very important to limit human exposure to wildlife to reduce risk of CoV transmission from animals to humans.

## Data Availability

All data are contained in this manuscript, its references and accession numbers mentioned and hence freely available.
